# Molecular Phylogenetics and Historical Biogeography of Subtribe Ecliptinae (Asteraceae, Heliantheae)

**DOI:** 10.3390/plants13192817

**Published:** 2024-10-08

**Authors:** Rafael Felipe de Almeida, Maria Alves, Cássio van den Berg, Marco O. O. Pellegrini, Morgan R. Gostel, Nádia Roque

**Affiliations:** 1 Royal Botanic Gardens Kew, Richmond, Surrey TW9 3AE, UK; r.felipe_de_almeida@kew.org (R.F.d.A.); m.pellegrini@kew.org (M.O.O.P.); 2 Departamento de Ciências Biológicas, Universidade Estadual de Feira de Santana, Feira de Santana 44036-900, Bahia, Brazil; mariaalvescompositae@gmail.com (M.A.); vcassio@gmail.com (C.v.d.B.); 3 Botanical Research Institute of Texas, Fort Worth, TX 76107-3400, USA; mgostel@brit.org; 4 Department of Botany, National Museum of Natural History, MRC 166, Smithsonian Institution, Washington, DC 20013-7012, USA; 5 Instituto de Biologia, Universidade Federal da Bahia, Salvador 40170-110, Bahia, Brazil

**Keywords:** compositae, dry forests, savannas, South America, systematics, taxonomy

## Abstract

We present a molecular phylogeny for the subtribe Ecliptinae (Asteraceae, Heliantheae) based on three plastid (*matK*, *psbA-trnH*, and *trnQ-rps16*) and two nuclear (nrITS and nrETS) markers. The results of the phylogenetic reconstruction were utilised as a topological constraint for a subsequent divergence dating analysis and ancestral range reconstructions. We sampled 41 species and 40 genera (72%) of Ecliptinae and two species of *Montanoa* (as outgroups) to elucidate the generic relationships between the genera of this subtribe. The Bayesian inference (BI) and Maximum Likelihood (ML) analyses were performed for the combined molecular dataset. The divergence dating analysis was performed using a relaxed, uncorrelated molecular clock with BEAST v1.8.4 and calibrated using a single secondary calibration point from a recently published chronogram for the family. The ancestral range reconstructions focusing on continents (i.e., South America, North America, Africa, Asia, and Oceania) and biomes (Dry forests, Altitudinal grasslands, Savannas, and Rainforests) were performed on BioGeoBEARS. Our phylogenetic results indicate that the genera of Ecliptinae are grouped into five clades, informally named the *Monactis*, *Oblivia*, *Blainvillea*, *Wedelia*, and *Melanthera* clades. The most recent, common ancestor of Ecliptinae was widespread in the North and South American dry forests at 8.16 Ma and mainly radiated in these regions up to the Pleistocene. At least eight dispersal events to South America and four dispersal events from North America to Africa, Asia, and Oceania took place during this period in all five informal clades of Ecliptinae. At least 13 biome shifts from dry forests to rainforests were evidenced, in addition to ten biome shifts from dry forests to altitudinal grasslands and savannas. These results corroborate the mid-late Miocene to early Pleistocene radiation of Ecliptinae in tropical dry forests. Future studies should aim to sample the remaining 14 unsampled genera of Ecliptinae to position them in one of the five informal clades proposed in this study.

## 1. Introduction

Heliantheae (Asteraceae) is one of the 13 tribes currently accepted in the Heliantheae alliance, with 113 genera and ca. 1500 species mostly confined to the Neotropics [1]. Most species are recognised by their opposite, trinerved leaves, radiate capitula, paleaceous receptacle, palea that usually conduplicate and surround the flowers, yellow corollas, strongly flattened cypselae, a pappus of persistent awns and squamellae disposed in a narrowly ovate to linear pattern on the cypsela neck, sometimes reduced to an erose crown, and a prominent layer of phytomelanin in the cypselae [2,3,4]. This tribe is one of the most economically important in the Compositae family due to its placement of the sunflower (*Helianthus annuus* L.), which is notable for its agricultural and ornamental use, as well as several commonly cultivated garden species from the genera *Echinacea* Moench, *Rudbeckia* L. and *Zinnia* L. [1]. Heliantheae currently comprises 15 accepted subtribes [5] that have been the subject of several molecular phylogenetic studies throughout the last two decades [6,7,8,9,10]. Despite the efforts of recent molecular phylogenetic studies, several subtribes remain problematic due to large polyphyletic genera such as *Melanthera* Rohr and *Wedelia* Jacq. [7].

Among the problematic subtribes of Heliantheae, Ecliptinae Less. is notable as it includes the aforementioned genera and 53 others (Supplementary Material S1) and includes 575 pantropical species, which are naturally absent from Europe [3,11]. Several studies have focused on understanding the generic relationships in the subtribe Ecliptinae throughout the last two decades [6,10,12,13,14]. Panero et al. [6] used 16 restriction endonuclease sites and recovered 27 genera of Ecliptinae as monophyletic, sisters to *Montanoa*, and divided them up into three main clades: *Monactis*, *Oblivia*, and *Eclipta* clades. These clades were recovered and well supported by statistical analyses, but the relationships within the genera placed in these clades were mostly weekly supported. Moraes et al. [10] sampled seven plastid markers (*matK, petB, petD, ndhI-ndhG, trnT-L, trnL-F*, and *rpl20-rps12*) for 33 genera of Ecliptinae, recovering four main clades in this subtribe: *Monactis*, *Oblivia*, *Blainvillea*, and *Eclipta* clades. Once more, these clades were recovered and well supported by statistical analyses, but the relationships within their genera and among these clades were mostly weekly supported.

Moraes et al. [12] sampled two nuclear markers (ETS and ITS) for 34 genera of Ecliptinae, recovering the same four clades from their previous study using just plastid markers. Ren [13] sampled ten plastid (5′*trnK* intron, *matK* gene, *trnT-trnL* spacer, *trnL* intron, *trnL-F* spacer, *rbcL-accD* spacer, *psbH-petB* spacer, *petB* intron, *petB-petD* spacer, and *petD* intron) markers and 33 genera of Ecliptinae to propose a new genus, *Indocypraea* Orchard, segregated from *Wollastonia* DC. ex Decne. This author recovered the clades *Monactis*, *Oblivia*, *Blainvillea*, and *Eclipta* as monophyletic but failed to sample any members of the *Melanthera* clade. Finally, Edwards et al. [14] sampled two nuclear (ETS and ITS) and two plastid (*psbA-trnH* and *trnQ-rps16*) markers for 19 genera of Ecliptinae but focused on the *Melanthera* clade (referenced by them as the *Melanthera* alliance). These authors aimed to test the generic boundaries and biogeographic hypotheses in the lineages of this clade, but a biogeographic analysis remained elusive due to the lack of a dated and calibrated phylogeny and ancestral range estimates.

We present a molecular phylogenetic study for Ecliptinae sampling two nuclear (ETS and ITS) and three plastid (*matK*, *psbA-trnH*, and *trnQ-rps16*) markers for 40 of the 55 (72%) currently accepted genera in this subtribe as a baseline to further explore the relationships among its genera. We also estimate the divergence dates for this subtribe and use the obtained time tree to perform an ancestral range reconstruction for both the continental and biome distributions and to elucidate its historical biogeography worldwide.

## 2. Materials and Methods

### 2.1. Taxon Sampling and Molecular Protocols

We sampled 43 taxa, including outgroups (*Montanoa hibiscifolia* Benth. and *M. karvinskii* DC.), representing 41 species and 40 genera of Ecliptinae (out of a total of 55 genera; Supplementary Material S2). Leaf material was sampled from silica-gel-preserved or herbarium specimens (12–80 mg) and for DNA extraction. Genomic DNA was extracted using the CTAB 2× protocol, modified by Doyle and Doyle [15]. Fragments from five loci were amplified by a Polymerase Chain Reaction (PCR). Three plastid (*matK*, *psbA-trnH*, and *trnQ-rps16*) and two nuclear regions (nuclear ribosomal ITS and ETS) were targeted based on their variability and the number of informative characters in Ecliptinae [6,10,12,13,14]. Primers and protocols used to amplify and sequence all regions were the same as those used by Moraes et al. [12] and Edwards et al. [14]. The amplification mix that achieved success for all regions was a TopTaq (QIAGEN, Hilden, Germany) mix following the standard protocol provided by the manufacturer, with the addition of 1.0 M betaine and 2% DMSO for the nrETS and nrITS regions [16]. PCR products were purified using PEG 11% precipitation (polyethylene glycol) [16] and were sequenced directly with the same primers used for the PCR amplification, except for the ITS region, in which we used primers 92 and ITS4. Sequence chromatograms were produced in an automatic sequencer (ABI 3130XL Genetic Analyzer, Thermo Fischer, Waltham, MA, USA) using Big Dye Terminator 3.1 (Applied Biosystems, Thermo Fischer, Waltham, MA, USA). All newly generated sequences were submitted to GenBank (NCBI). Sequences had their ends trimmed using Geneious 4.84 [17] and aligned using Muscle v3 [18], with subsequent adjustments in the preliminary matrices made manually by eye. The complete data matrices are available in Supplementary Material S3.

### 2.2. Phylogenetic Analyses

All trees were rooted with the genus *Montanoa* (Montanoinae) following Edwards et al. [14]. The model of nucleotide evolution was selected using hierarchical likelihood ratio tests, using the J Modeltest 2 [19]. The Bayesian inference (BI) and Maximum Likelihood analyses were conducted with a mixed model and unlinked parameters using MrBayes 3.1.2 [20]. The Markov Chain Monte Carlo (MCMC) was run using two simultaneous independent runs with four chains each (one cold and three heated), saving one tree every 1000 generations for a total of ten million generations. We excluded 25% of the retained trees as ‘burn-in’ and checked for a stationary phase of likelihood, checking for ESS values higher than 200 for all the parameters on the Tracer 1.6 [21]. The posterior probabilities (PP) of clades were estimated from a majority rule consensus, using the remaining trees (after burn-in), and calculated with MrBayes 3.1.2 [20]. The Maximum Likelihood analysis (ML) was performed with ten independent replicates, and default settings and support values were estimated using nonparametric bootstrapping (BS) with 500 replicates using RAxML v.8 [22] implemented in RAxMLGUI2 [23]. Character coding followed Sereno’s recommendations for morphological analyses [24]. Primary homology hypotheses [25] were proposed for habit, leaf, inflorescence architecture, floral, and fruit characters presented by Panero [3]. A total of 23 characters were scored and coded (Supplementary Material S4). All characters were optimised on the concatenated tree with the Maximum Likelihood function (mk1 model) using Mesquite 2.73 [26] and visualised on Winclada [27].

### 2.3. Calibration

Divergence dating estimates were conducted using BEAST 1.8.4 [28] based on the results using the Bayesian combined (five loci) tree generated by MrBayes as a topological constraint. The beast analysis parameters included a relaxed uncorrelated lognormal clock and Yule process speciation [28]. The calibration parameters were based on previous estimates derived from a comprehensive study of the whole Asteraceae [29]. We used a single calibration point in the most recent common ancestor (MRCA) of the subtribes Ecliptinae + Montanoinae using a normal prior (mean = 15.52 Mya and standard deviation = 1.0). Two separate and convergent runs were conducted, with 10,000,000 generations, sampling each 1000 steps and 2000 trees after burn-in. We checked for ESS values higher than 200 for all parameters on Tracer 1.6 [21]. Tree topology was assessed using the TreeAnnotator and FigTree 1.4.0 [30].

### 2.4. Ancestral Range Reconstruction

Species distribution data were compiled from POWO [11] and herbarium collections from specimens in the Global Biodiversity Information Facility (GBIF) [31]. Ancestral range reconstructions were defined for the continents (A—South America, B—North America, C—Africa, D—Asia, E—Oceania) and biomes (A—Dry forests, B—Altitudinal grasslands, C—Savannas, D—Rainforests). Ancestral ranges of Ecliptinae were estimated using the R package BioGeoBEARS version 1.1.1 [32] and the models DEC, DIVALIKE, and BAYAREALIKE. Considering the possibility of underestimation of extinction rates due to the null ranges of the species allowed by the models, analyses with the “*” option were also performed. The “j” option was used to consider founder speciation events. Different combinations of the three models and the two options generated a total of 12 models. The best-fitting models for analyses were determined by AIC weight ratios.

## 3. Results

### 3.1. Phylogenetics of Ecliptinae

**Figure 1:**
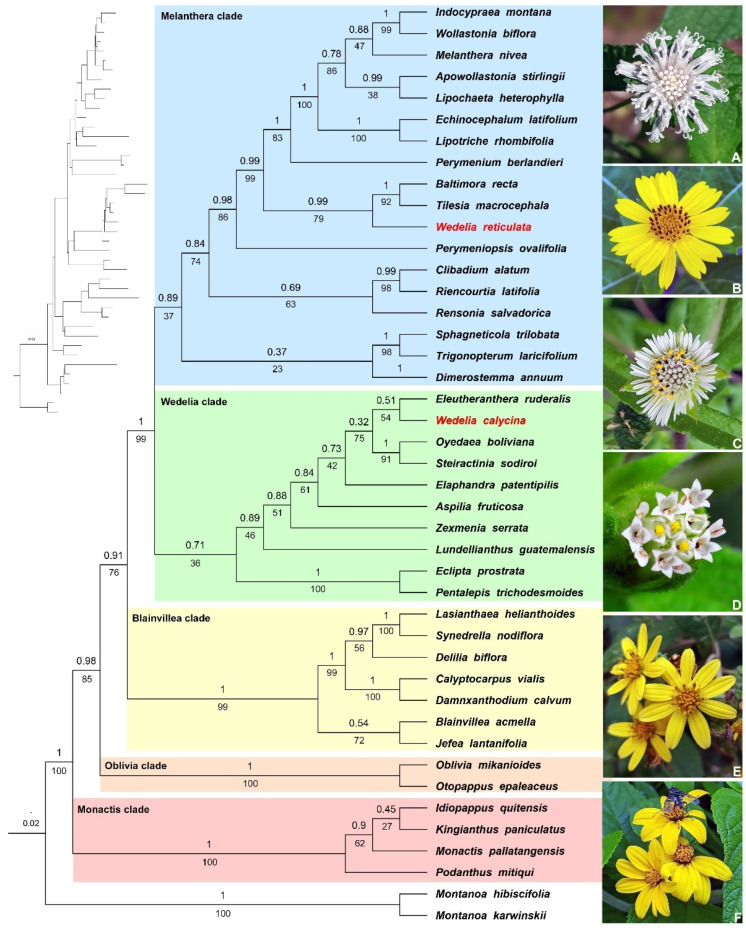
Molecular phylogeny of Ecliptinae, highlighting the five informal clades proposed here: right tree shows posterior probabilities of the Bayesian inference shown above branches and bootstrap values shown below branches, and left tree shows branch lengths. (**A**). *Melanthera nivea* (L.) Small by Katie Z, (**B**). *Wedelia calycina* Rich. by Juan Gabriel, (**C**). *Eclipta prostrata* (L.) by Nana Ten, (**D**). *Blainvillea gayana* Cass. by Frederico Acaz Sonntag, (**E**). *Otopappus verbesinoides* Benth. by Luis Humberto Vicente-Rivera, and (**F**). *Kingianthus paniculatus* (Turcz.) H.Rob. by Yanna Paola.

The nuclear dataset represented 1317 characters, the plastid dataset represented 3892 characters, and the combined plastid + nuclear (5 loci) dataset included 5209 analysed characters (Supplementary Material S2). The topologies produced by the BI and ML based on the individual nuclear and plastid datasets were evidently congruent, so we performed a combined analysis of the plastid + nuclear datasets. Both the BI and ML analyses recovered a fully resolved tree with mostly well-supported clades (>PP 0.95/BS 70%; Figure 1). The results suggest that the genera of Ecliptinae were recovered in five informal clades, which we have referred to as the *Monactis*, *Oblivia*, *Blainvillea*, *Wedelia*, and *Melanthera* clades (Figure 1). Among each of these clades, the *Monactis* clade was recovered with strong branch support (PP1/BS100%) as a sister to the rest of Ecliptinae and comprises the genera *Idiopappus* H.Rob. & Panero, *Kingianthus* H.Rob., *Monactis* Kunth, and *Podanthus* Lag. (Figure 1). The *Oblivia* clade was well supported (PP1/BS100%) as a sister to the *Blainvillea*, *Wedelia*, and *Melanthera* clades and comprises the genera *Oblivia* Strother and *Otopappus* Benth. (Figure 1). The *Blainvillea* clade is also well supported (PP1/BS99%) and comprises seven genera: *Blainvillea* Cass., *Calyptocarpus* Less., *Damnxanthodium* Strother, *Delilia* Spreng., *Jefea* Strother, *Lasianthaea* DC., and *Synedrella* Gaertn. (Figure 1). The *Wedelia* clade was only moderately to weakly supported (PP0.71/BS36%) and comprises several genera, including *Aspilia*, *Eclipta* L., *Elaphandra* Strother, *Eleutheranthera* Poit., *Lundellianthus* H.Rob., *Oyedaea* DC., *Pentalepis* F.Muell., *Zexmenia* La Llave, and *Wedelia* (Figure 1). Last, the *Melanthera* clade was only moderately supported (PP0.89/BS37%) and comprises the remaining 18 genera sampled in this study (i.e., *Apowollastonia* Orchard, *Baltimora* L., *Clibadium* F.Allam. ex L., *Dimerostemma* Cass., *Echinocephalum* Gardner, *Indocypraea* Orchard, *Lipochaeta* DC., *Lipotriche* R.Br., *Melanthera* Rohr, *Perymeniopsis* H.Rob., *Perymenium* Schrad., *Rensonia* S.F.Blake, *Riencourtia* Cass., *Sphagneticola* O.Hoffm., *Tilesia* G.Mey., *Trigonopterum* Steetz, *Wedelia*, and *Wollastonia* DC. ex Decne.). The 18 genera that comprise the *Melanthera* clade correspond to the eight moderately- to well-supported subclades (Figure 1). *Wedelia reticulata* DC. was also recovered in the *Melanthera* clade in a subclade with *Baltimora* and *Tilesia* with strong branch support (PP0.99/BS79%), making *Wedelia* paraphyletic (Figure 1).

**Figure 2:**
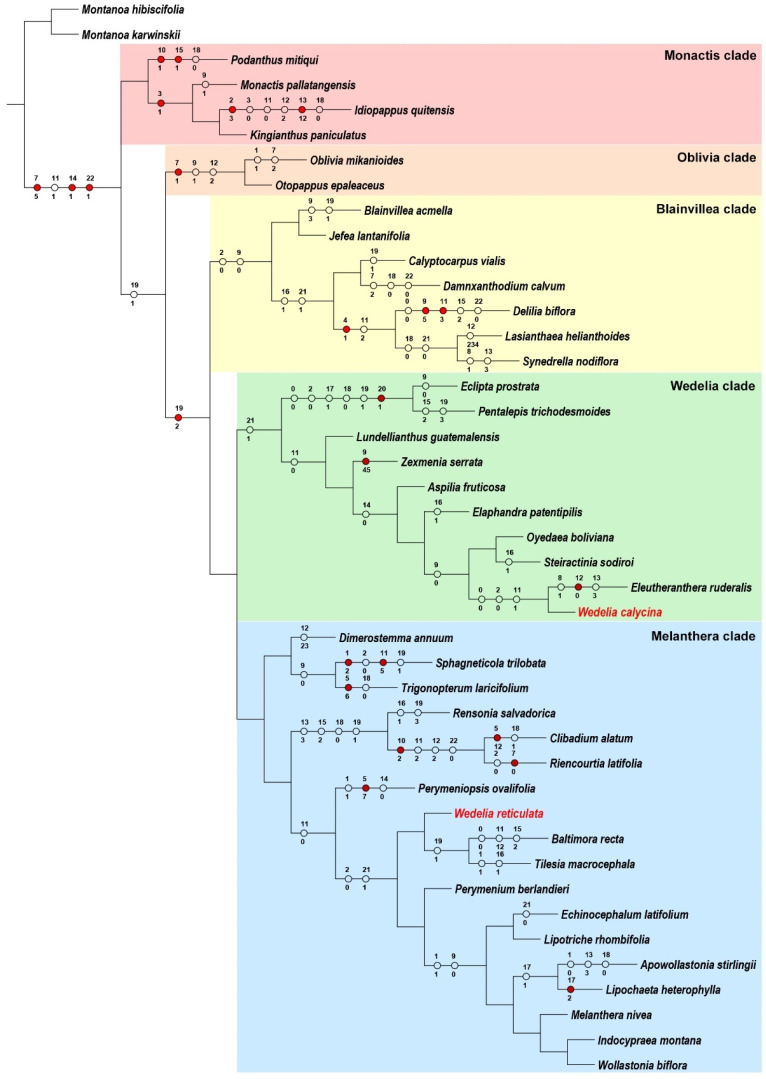
Character-mapping of 23 morphological traits in Ecliptinae, highlighting the five informal clades proposed here. Red circles represent apomorphies. Transparent circles represent homoplasies. Numbers above circles represent character numbers, and numbers below circles represent character states.

Ecliptinae was recovered and supported by three synapomorphies (3-plinerved leaf blades, ray florets pistillate, and cypselae with pappus) and a single homoplasy (capitula with involucres campanulate, Figure 2). The *Monactis* clade had no morphological traits recovered in this analysis, but the *Oblivia* + *Blainvillea* + *Wedelia* + *Melanthera* clade was recovered and supported by a single homoplasy (gynoecium with style arms tapered at apex; Figure 2). The *Oblivia* clade was recovered and supported by a single synapomorphy (2–5-plinerved leaf blades) and two homoplasies (corymbiform inflorescences and capitula with phyllaries in three series, Figure 2). The *Blainvillea* + *Wedelia* + *Melanthera* clade was recovered and supported by a single synapomorphy (gynoecium with style arms tapered and papillose at the apex, Figure 2). The *Blainvillea* clade was recovered and supported by two homoplasies (herbs and solitary capitula), while the *Wedelia* + *Melanthera* clade had no morphological traits recovered in this analysis (Figure 2). The *Wedelia* clade was recovered and supported by a single homoplasy (hairy cypselae), while the *Melanthera* clade had no morphological traits recovered in this analysis (Figure 2). It is worth mentioning that *Wedelia* s.l. (*Aspilia* + *Elaphandra* + *Oyedaea* + *Steiractinia* + *Eleutheranthera* + *Wedelia* clade) was recovered and supported by a single homoplasy (ray florets neuter), while *Wedelia reticulata* was recovered and found to be distantly related to the *Melanthera* clade (Figure 2).

### 3.2. Divergence Times and Ancestral Ranges of Ecliptinae

**Figure 3:**
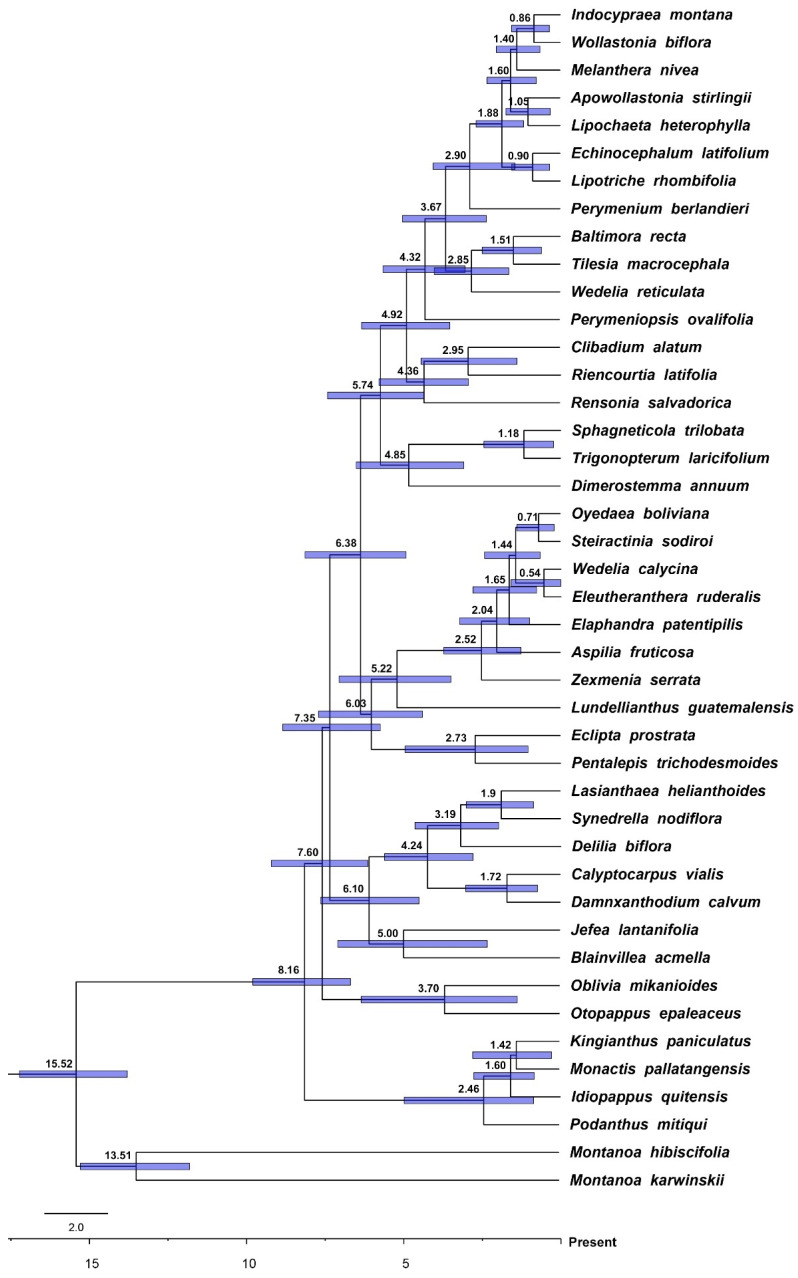
Chronogram of Ecliptinae showing mean node ages estimated for branches. Blue bars represent 95% Highest Posterior Densities (HPD) for the estimated median dates.

**Figure 4:**
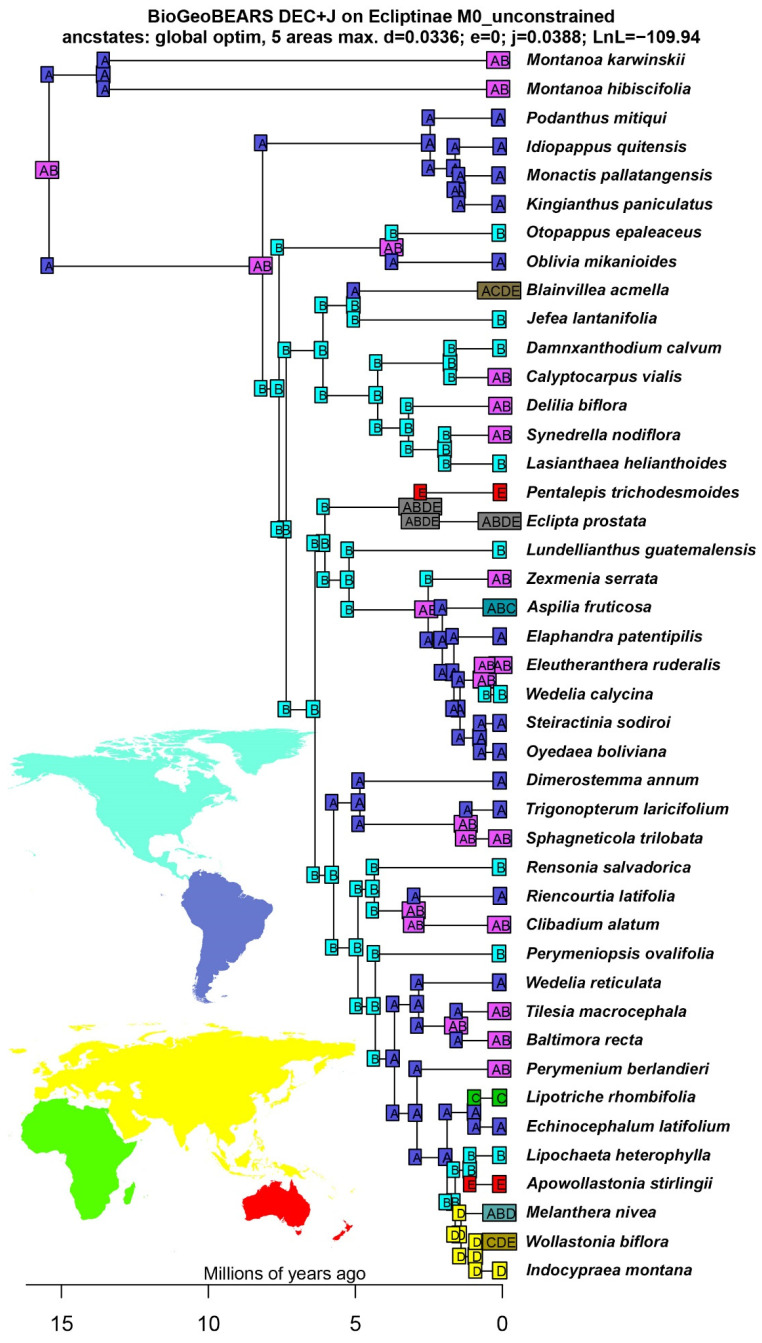
Continental ancestral range reconstruction for Ecliptinae: (**A**) South America, (**B**) North America, (**C**) Africa, (**D**) Asia, and (**E**) Oceania.

**Figure 5:**
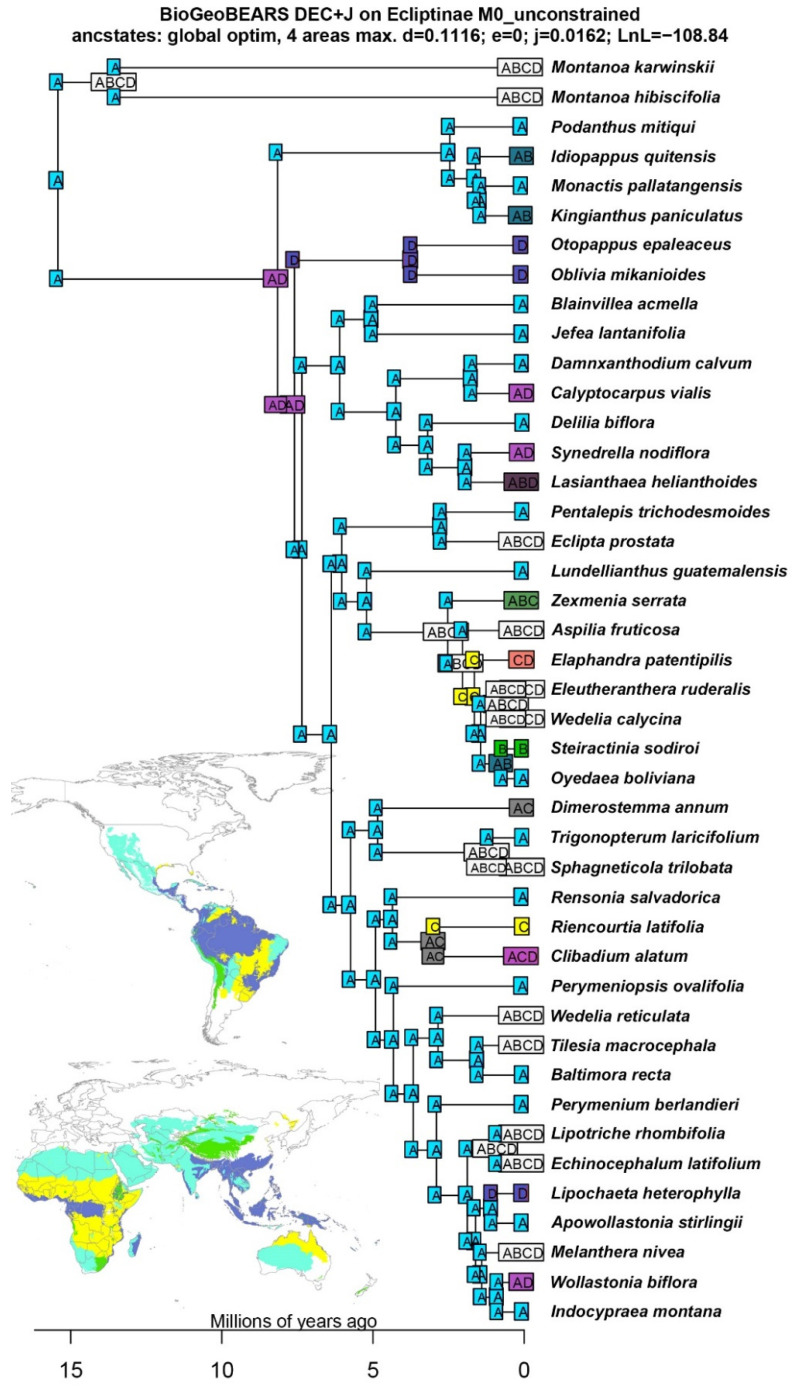
Biome ancestral range reconstruction for Ecliptinae: (**A**) Dry forests, (**B**) Altitudinal grasslands, (**C**) Savannas, and (**D**) Rainforests.

The estimates of divergence times and ancestral range reconstructions show that the most recent common ancestor (MRCA) of Ecliptinae dates back to 8.16 Ma (9.80–6.69 Ma 95% HPD, Figure 3) and was widespread in the dry forests and rainforests in North and South America (Figure 4 and Figure 5). The MRCA of the *Monactis* clade arose at 2.46 Ma in the dry forests of South America, more specifically, those along the Andes Mountain range. This clade also underwent two different biome shifts to altitudinal grasslands in *Idiopappus* and *Kingianthus* from 1.60 to 1.42 Ma. The MRCA of the *Oblivia* clade emerged at 3.70 Ma in the rainforests of both North and South America, with *Oblivia* radiating exclusively in South America and *Otopappus* exclusively in North America. The MRCA of the *Blainvillea* clade arose at 6.10 Ma in the dry forests of North America, with three recolonisation events in South America’s dry forests and rainforests from 3.19 to 1.72 Ma. The MRCA of the *Wedelia* clade emerged at 6.03 Ma in the dry forests of North America, including the first two dispersal events from North America to Oceania (i.e., Australia) at 2.73 Ma and to Africa at 2.04 Ma. This clade also comprises the first colonisation event of the South American savannas at 2.52 Ma in the MRCA of all the genera related to *Wedelia* s.s. Finally, the *Melanthera* clade arose at 5.74 Ma in the dry forests of North America. This clade also comprises three different recolonisation events in South America at 4.85, 3.67, and 2.95 Ma, the first colonisation events in Africa at 0.90 Ma and Asia at 1.40 Ma, and the second dispersal event from North America to Oceania (i.e., Australia) at 1.05 Ma. And, regarding biomes, the *Melanthera* clade shows the second colonisation event of the South American savannas at 2.95 Ma (Figure 3, Figure 4 and Figure 5).

## 4. Discussion

### 4.1. Phylogenetics and Systematics of Ecliptinae

The five main clades of Ecliptinae recovered in this study have consistently been recovered by all previous molecular phylogenetic studies on Heliantheae [6,10,12,13,14]. Additionally, the morphological traits used by Panero [3] in the last taxonomic treatment for this subtribe were recovered as homoplasies and synapomorphies supporting *Ecliptinae*, *Oblivia*, *Blainvillea*, and *Eclipta* clades. Our results show that the generic circumscriptions in Ecliptinae are affected mainly by the non-monophyly of two genera, *Melanthera* (six species) and *Wedelia* (131 species). Edwards et al. [14] showed that *Melanthera latifolia* (Gardner) Cabrera was recovered as a sister to *Lipotriche* in their analyses, making *Melanthera* paraphyletic. To solve this matter, these authors formally proposed the reestablishment of *Echinocephalum* Gardner. In our results, *Wedelia calycina* Rich. (i.e., *Wedelia* s.s.) was placed in the *Wedelia* clade alongside several genera with neuter ray florets, such as *Aspilia*, *Elaphandra*, *Eleutheranthera*, *Oyedaea*, and *Steiractinia*. However, *Wedelia reticulata* DC. was recovered in the *Melanthera* clade, which corroborates evidence from previous studies on Ecliptinae [12,14] that indicate *Wedelia* is paraphyletic.

Historically, studies on the tribe Heliantheae show that cypselae provide good diagnostic morphological characters for generic recognition [3,33,34,35,36]. Although many of these characters were shown to be homoplasic in Ecliptinae lineages, according to previous studies [10,37] and this current one, cypselae morphology among the genera in the *Wedelia* clade might also be of taxonomic relevance when circumscribing the genera in this group. Most genera in this clade have dimorphic cypselae in the same head when the ray florets are fertile, with coroniform-aristate or aristate pappus above the rostrum, with elaiosomes or scars left by them [3,36]. Future molecular studies will have to include a better sampling of the species of *Wedelia s.s.* and the related genera besides the 14 mostly monotypic genera not currently sampled in this study (i.e., *Exomiocarpon* Lawalrée, *Fenixia* Merr., *Hoffmanniella* Schltr. ex Lawalrée, *Iogeton* Strother, *Lantanopsis* C.Wright ex Griseb., *Leptocarpha* DC., *Pascalia* Ortega, *Plagiolophus* Greenm., *Schizoptera* Turcz., *Synedrellopsis* Hieron. & Kuntze, *Tuberculocarpus* Pruski, *Tuxtla* Villaseñor & Strother, *Wamalchitamia* Strother, and *Zyzyxia* Strother) to properly shed some needed light on this matter. Furthermore, future studies should aim to sample a more significant number of sequence loci, particularly as previous studies have indicated an incongruence and the suggested incongruence between the nuclear and plastid data might have resulted from ancestral hybridisation events and/or polyploidisation [38]. The extent and role of hybridisation and polyploidisation should be more carefully and exhaustively explored before significant and consequential taxonomic changes are proposed to the generic circumscription of Ecliptinae.

### 4.2. Historical Biogeography of Ecliptinae

The diversification of Ecliptinae in continents and biomes seems to corroborate other patterns of biome diversification in the Neotropics. Our results suggest the MRCA of Ecliptinae and all its informal groups (i.e., *Monactis*, *Oblivia*, *Blainvillea*, *Wedelia*, and *Melanthera* clades) radiated in North American dry forests between the end of the Miocene and the start of the Pleistocene from a South American ancestor. This pattern is also well documented in other flowering plant groups, such as *Verbesina* L. (Verbesininae Benth., Asteraceae), *Stevia* Cav., and Malpighiaceae Juss. In *Verbesina*, the MRCA of this genus radiated in North American dry forests at the end of the Miocene, with two posterior dispersal events in South American dry forests in the Pleistocene [39]. In *Stevia*, the MRCA of this genus originated in Mexico at 7.0–7.3 Ma, probably in pine–oak forests and the dispersion to South America occurred only a single time at 4.5 Ma [40]. On the other hand, Malpighiaceae shows at least 33 colonisation events from South to North America during the same geological time [41].

Since most dispersal events in Ecliptinae from South to North America happened after 10 Ma, a scenario of long-distance dispersal (or jump dispersal) cannot be invoked to explain the colonisation and radiation of most lineages of this subtribe in North America. The Panama Isthmus started forming at 10 Ma when the Central American Seaway (CAS, defined as the ocean gap along the tectonic boundary between the South American plate and the Panama microplate) was closed [42]. From 10.0 to 3.5 Ma, there were intermittent Caribbean–Pacific connections through pathways other than the CAS. At 3.5 Ma, the Isthmus was completely closed [42]. Additionally, the only two clades that represent members of this subtribe from savannas also radiated from 2.95 to 2.04 Ma in the Pleistocene, corroborating the mid-Miocene to Pleistocene diversification of the savannic lineages of South America [43,44].

The MRCA of Ecliptinae and all its main lineages have arisen and radiated in the American continent from 8.17 to 0.90 Ma, with few long-distance dispersal events recorded on different continents within this timeframe. Our results evidence three different Pleistocene patterns of long-distant dispersals from North America to Oceania (2.73 to 1.05 Ma in *Pentalepis*/*Eclipta* and *Apowollastonia*) and Asia (0.86 Ma in *Wollastonia*/*Indocypraea*), and from South America to Africa (0.90 Ma, in *Lipotriche*). Long-distant dispersal events from the American continent to elsewhere are well documented in the literature from the Eocene to the Miocene [45,46]. Still, Pleistocene dispersal events from the Americas to Africa, Asia, and Oceania seem to be understudied in the historical biogeography literature. Pleistocene glaciations seem to be one of the few explanations for these long-distance dispersal events in Ecliptinae. During this period, sea levels diminished, and several land bridges were connecting proximately related landmasses [47,48].

## 5. Conclusions

Our study is the first to test the relationship of 40 genera (out of 55) of Ecliptinae, and it is the first time that *Aspilia* was sequenced. Evidence from recent studies suggest the widespread paraphyly of two genera in this subtribe, including *Melanthera* (six species) and *Wedelia* (131 spp.), and question the implications of these findings in guiding future taxonomic re-circumscription in Ecliptinae. Five main clades were recovered and supported by the molecular and morphological data: the *Monactis*, *Oblivia*, *Blainvillea*, *Wedelia*, and *Melanthera* clades. Our findings corroborate those from previous studies that indicate *Wedelia* is not monophyletic. However, we caution against any significant taxonomic changes until additional sampling (both taxa and molecular loci) is available. The MRCA of Ecliptinae seems to have arisen at 8.16 Ma and diversified in North and South America up to the Pleistocene. At least eight dispersal events to South America and four dispersal events from North America to Africa, Asia, and Oceania took place during this period in all five informal clades of Ecliptinae. At least 13 biome shifts from dry forests to rainforests and ten biome shifts from dry forests to altitudinal grasslands and savannas took place in this subtribe. An increased sampling and further molecular markers are still needed for a better understanding of the morphological evolution and diversification of this subtribe.

## Data Availability

All data used in this study are available in Supplementary Materials S1–S4.

## Supplementary Materials

The following supporting information can be downloaded at https://www.mdpi.com/article/10.3390/plants13192817/s1, Supplementary Materials S1–S4.
